# Recurrence of intraparotid facial nerve schwannoma: A case report

**DOI:** 10.1016/j.bjorl.2026.101904

**Published:** 2026-10-05

**Authors:** Fábio Coelho Alves Silveira, Camila Barbosa Marinho Muniz, Maria Clara Peregrino Torres Vieira de Melo, José Miguel Falcão Rodrigues Sampaio, Thiago Victor Leite Aquino, Caio Vinicius Ferreira da Silva Andrade, Layla Carvalho Mahnke

**Affiliations:** a Universidade Federal de Pernambuco (UFPE), Hospital das Clínicas, Departamento de Otorrinolaringologia, Cirurgia de Cabeça e Pescoço, Recife, PE, Brazil; b Universidade de Pernambuco (UPE), Faculdade de Ciências Médicas, Recife, PE, Brazil; c Fundação Altino Ventura (FAV), Recife, PE, Brazil

**Keywords:** Facial nerve neoplasms, Fine-Needle aspiration, Nerve transfer techniques, Neurogenic tumors, Peripheral faciam paralysis

## Introduction

Schwannomas are typically benign, slow-growing tumors originating from Schwann cells of peripheral nerves that may cause neurological symptoms due to nerve compression.^1^^,^^2^

Facial Nerve Schwannomas (FNS) are uncommon, and present with facial paralysis, hearing loss or a regional mass.^1^ Those arising in the intraparotid segment are extremely rare and often preserve facial nerve function.^2^ This case highlights the recurrence of an Intraparotid Facial Nerve Schwannoma (IFNS) with worsening clinical presentation after debulking, an aspect rarely addressed in current literature, and discusses diagnostic and therapeutic challenges.

## Case report

We present a case of a recurrent IFNS in a 58-year-old mixed-race Brazilian woman. The patient’s medical history included hypertension treated with propranolol and tubal ligation, with no other comorbidities, allergies, or drug use.

The patient presented with isolated facial paralysis and was evaluated by an otolaryngologist at a regional public referral hospital. Physical examination revealed grade IV Peripheral Facial Paralysis (PFP) on the House-Brackmann scale. Magnetic resonance imaging (MRI) revealed an expansile, contrast-enhancing lesion with diffusion restriction along the right facial nerve, with an intraparotid component measuring 1.9 × 1.6 × 1.5 cm (Fig. 1).

**Fig. 1:**
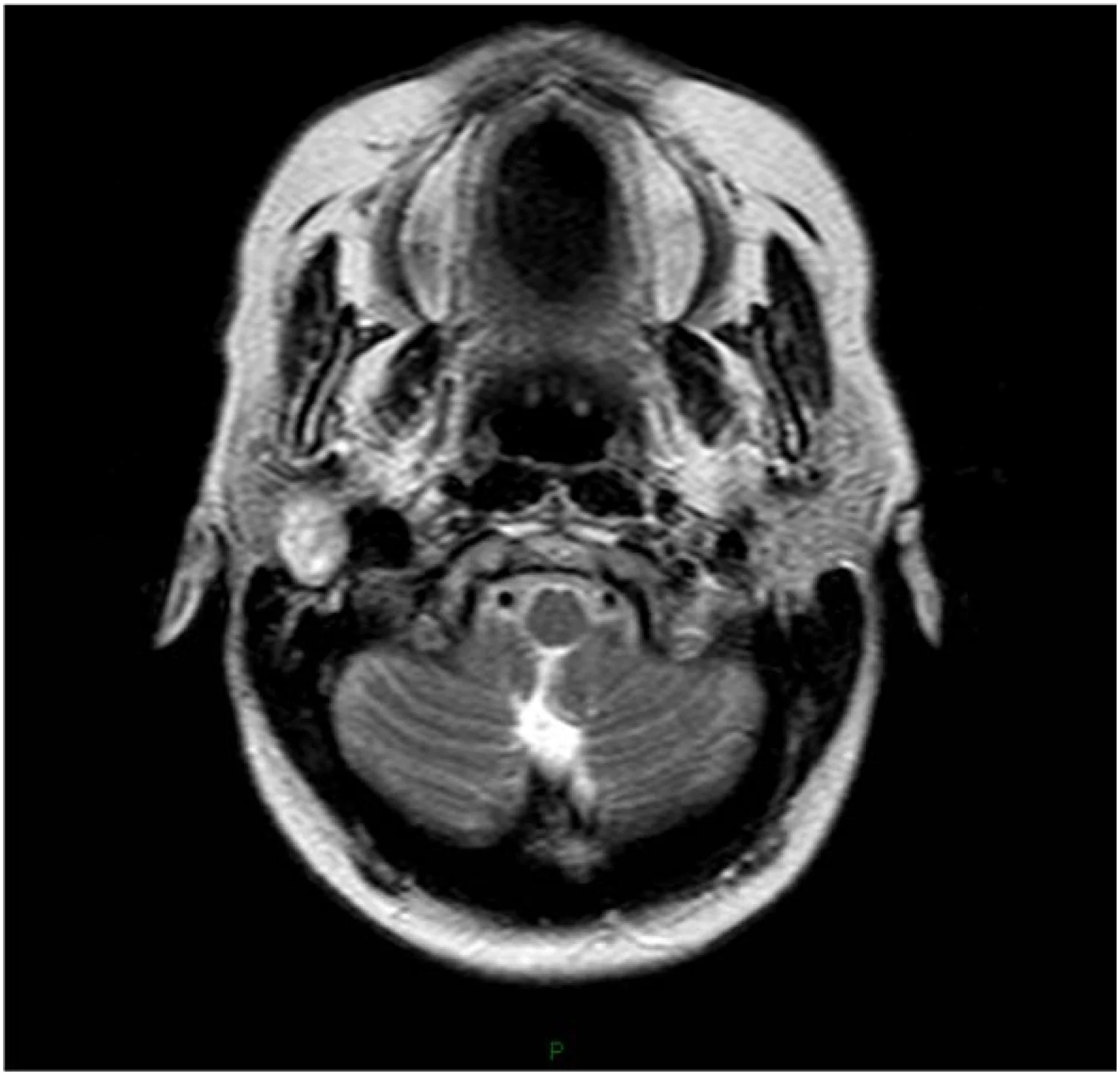
MRI revealing an intraparotid mass following the right facial nerve.

Fine-Needle Aspiration (FNA) of the intraparotid nodule was performed. Cytology showed fibromyxoid stroma with cells displaying scant cytoplasm, wavy nuclei with tapered ends and occasional intranuclear incursions. One mitotic figure and focal nuclear palisading were observed. Findings were consistent with schwannoma.

Surgical management consisted of parotid tumour resection. A preauricular incision with cervical extension was performed, followed by elevation of skin flaps and exposure of the parotid gland. The main trunk of the facial nerve was not identified. A cystic lesion enveloping the facial nerve was visualized.

Histopathological examination confirmed schwannoma. Due to the low reported recurrence of cranial schwannomas, tumor debulking with facial nerve preservation was performed. Intraoperatively, thinning of the facial nerve fibers was observed following lesion reduction. Microsurgical repair was performed, and hemostasis achieved.

Postoperatively, the patient remained clinically stable with persistent grade IV PFP. Early wound evaluation was satisfactory, with unchanged PFP. Physiotherapy was initiated and lubricating eye drops were prescribed.

Two years and eight months later, Computed Tomography (CT) revealed a lobulated nodular lesion in the right parotid space, with heterogeneous contrast enhancement and measuring 1.5 × 1.4 cm. The patient also reported local pain and worsening PFP, which had progressed to grade V on the House-Brackmann scale. Conservative management with clinical monitoring and analgesics was instituted.

One and a half years later, paralysis progressed to grade VI. An MRI was requested, which was delayed 15-months because of limited access to specialized imaging. It demonstrated hypodense material in the right inner ear with extension to the parotid gland and similar characteristics to the prior study, with slight enlargement of the posterior parotid component measuring approximately 1.3 × 1.2 cm.

Given these findings, complete tumor resection with hypoglossal-facial nerve anastomosis was planned for facial reanimation.

## Discussion

The behaviour of FNS is largely determined by tumor topography.^1^ Intratemporal lesions predominate and characteristically produce facial nerve dysfunction, whereas the rare extratemporal tumours involving the intraparotid segment usually present as parotid masses with preserved facial function.^1^ Although most IFNS are described as indolent and slow growing, this case demonstrates that those tumors may follow an aggressive trajectory that the prevailing literature does not adequately capture.^1^

Diagnostic uncertainty reflects a limitation intrinsic to the entity. Cross-sectional imaging of cranial nerve schwannomas is frequently subtle and nonspecific, demanding clinicoradiologic correlation and detailed anatomical knowledge.^3^ MRI typically reveals heterogeneous T2 hyperintensity, low-to-intermediate T1 signal intensity and avid contrast enhancement.^3^ Although the “target sign” is considered specific for schwannoma, it is seldom observed in cranial-nerve tumors.^3^ Uncertainty is greater in intraparotid schwannomas, which usually present as circumscribed parotid masses and frequently mimic benign salivary neoplasms.^3^

Cytological assessment is similarly constrained. Fine-needle aspiration of peripheral nerve sheath tumours yields heterogeneous, frequently nonspecific findings that overlap with other spindle-cell neoplasms, while degenerative atypia may simulate malignancy and characteristic architectural features may be absent.^4^ Cytology is therefore prone to nondiagnostic or equivocal results, and ancillary immunohistochemistry is often required for definitive classification.^4^

The optimal surgical management of IFNS remains non-consensual because treatment must balance oncologic control with preservation of facial nerve function, a decision compounded by the frequent absence of definitive preoperative diagnosis.^2^^,^^5^ Radiologic surveillance is therefore limited to select patients, particularly elderly individuals in whom a confident diagnosis can be established.^5^

When surgery is undertaken, total resection is advocated when facial movement is already poor or the nerve cannot be preserved, mandating reconstruction. Nerve-sparing subtotal techniques are appropriate only when intraoperative findings permit fascicular preservation and are associated with superior functional outcomes.^2^^,^^5^

This case highlights the limitations of nerve-preserving surgery in patients with advanced facial dysfunction. Subtotal nerve-sparing resection was followed by recurrence within 3-years, pain, and paralysis progression, ultimately requiring complete resection with hypoglossal-facial anastomosis. The conservative strategy therefore did not prevent progression and arguably delayed definitive treatment.

Such an outcome is particularly instructive given the low reported recurrence rate of IFNS 2.38% (one patient in a 17-year cohort).^5^ The scarcity of documented recurrences makes the present case a meaningful illustration of treatment failure after initial nerve-preserving surgery, and supports a lower threshold for early radical resection in patients with substantial facial dysfunction.

## Conclusion

IFNS remains a rare entity associated with diagnostic uncertainty and complex therapeutic decision-making. This case illustrates that recurrence with progressive facial dysfunction may occur despite initial nerve-preserving management, underscoring the limitations of conservative or subtotal approaches in selected patients.

Careful clinicoradiologic evaluation and individualized surgical planning are therefore essential to optimize functional outcomes. These findings provide meaningful clinical insight to the limited literature on recurrent IFNS and its management.

## ORCID ID

Fábio Coelho Alves Silveira: 0009-0004-0906-9247

Camila Barbosa Marinho Muniz: 0000-0002-9464-133X

Maria Clara Peregrino Torres: 0000-0002-8954-9010

Jose Miguel Falcao Rodrigues Sampaio: 0000-0003-1990-722X

Thiago Victor Leite Aquino: 0009-0002-8850-9444

Caio Vinicius Ferreira da Silva Andrade: 0009-0009-9580-571X

Layla Carvalho Mahnke: 0000-0001-8758-0877

## Declaration of patient consent

The authors certify that they have obtained all appropriate patient consent forms. In the form, the patient has given her consent for her images and other clinical information to be reported in the journal. The patient understands that her name and initials will not be published, and due efforts will be made to conceal her identity, but anonymity cannot be guaranteed.

## Funding

This research did not receive any specific grant from funding agencies in the public, commercial or not-for-profit sectors.

## Data availability statement

The authors declare that all data are available in repository.

## Declaration of competing interest

The authors declare that they have no known competing financial interests or personal relationships that could have appeared to influence the work reported in this paper.
